# Myxoid Dermatofibrosarcoma Protuberans of the Abdomen

**Published:** 2016-01-04

**Authors:** Joshua B. Elston, Andrea Little, Brielle Weinstein, Kelly A. Segars, Paul D. Smith

**Affiliations:** ^a^Division of Plastic Surgery, Department of Surgery, University of South Florida Morsani College of Medicine, Tampa; ^b^Medical University of South Carolina College of Medicine, Charleston; ^c^Largo Medical Center, Largo, Fla; ^d^H. Lee Moffitt Cancer Center, Tampa, Fla

**Keywords:** dermatofibrosarcoma protuberans, DFSP, fibrosarcoma, abdominal reconstruction, malignant degeneration

## DESCRIPTION

A 60-year-old African American woman presented with a 5-year history of a large, left-sided abdominal mass originating in a remote burn scar. Resection was coordinated with the Sarcoma team, and reconstruction was performed with extensive mobilization and skin grafting. Pathology confirmed myxoid dermatofibrosarcoma protuberans (DFSP) with focal fibrosarcomatous changes, an extremely rare and malignant variant.

## QUESTIONS

1. **What are the classic etiological presentation features of DFSP?**
2. **What is the clinical course of DFSP?**
3. **What are accepted treatment options?**
4. **What are potential reconstructive options for this lesion?**

## DISCUSSION

DFSP is a rare dermal malignancy that accounts for less than 1% of soft-tissue sarcomas. It typically arises between the ages of 20 to 50 years, is slightly more common among men and African Americans, and usually appears as a dermal or subcutaneous multinodular tumor with surrounding violaceous skin changes on the trunk, head and neck, or extremities.^1^^,^^2^ DFSP may initially be misdiagnosed as a benign growth and only prompt biopsy when the tumor becomes troublesome to the patient.

DFSP is typically slow growing, which often delays the diagnosis. Dermal and subcutaneous finger-like projections often grow asymmetrically and well beyond the obvious clinical tumor.^1^^-^3 Despite this, clear margins has a favorable 5-year survival and disease-free rate above 95%.^2^^,^^4^ While most are low-grade and highly amenable to surgical treatment, a small percentage of lesions contain an element of high-grade sarcoma and have potential for distant metastasis.^1^^,^^3^ Larger lesions or those with biopsy-proven fibrosarcomatous changes may have a metastatic component of disease and consideration should be given to a staging workup.

In considering treatment options, a multidisciplinary team approach is mandatory. Surgical excision has been the gold standard. Mohs surgery permits tissue preservation and is preferable for smaller tumors but often impractical for larger tumors. Historically, recommended margins ranged from 3 to 5 cm. Recent evidence suggests that in-depth pathological analysis of all margins may be more important and margins of 1 to 2 cm are adequate.^2^^,^^5^ If inadequate margins could not be obtained because of the location near critical structures, a referral to Medical/Radiation Oncology may be warranted, although literature at this time is lacking.

**Figure 1:**
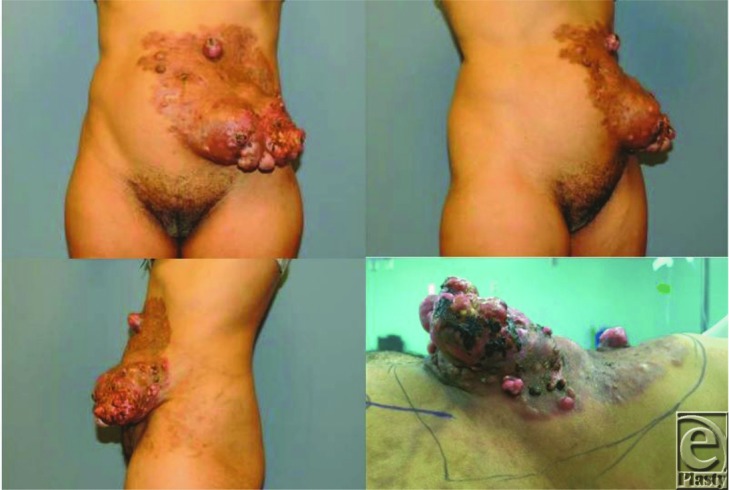
Advanced dermatofibrosarcoma protuberans of the abdominal wall. Preoperative photographs demonstrate the significance of the lesion, with the supine position being the most impressive to appreciate the fungating and necrotic nature of the mass.

**Figure 2:**
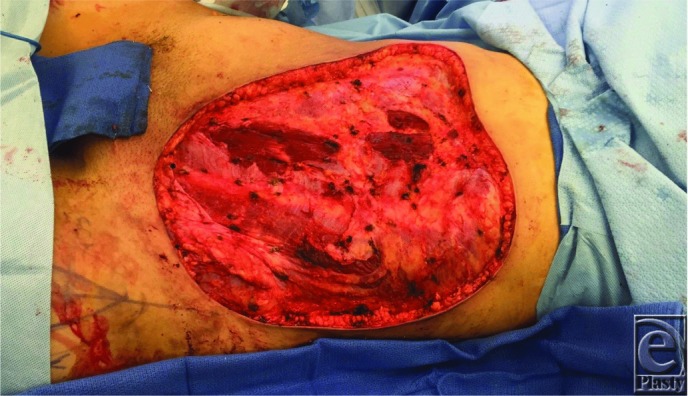
Intraoperative view of the large abdominal wall after resection by the Sarcoma team. Note the areas of anterior rectus sheath resected, although rectus abdominis remains intact.

**Figure 3:**
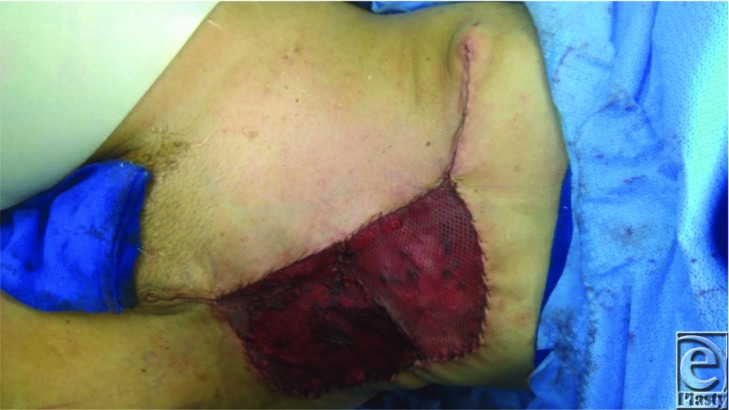
Despite anticipating the need for more extensive reconstruction preoperatively, the partial-thickness abdominal wall resection lent itself to mobilization of large adipocutaneous flaps and a resultant defect a third of the initial size. A split-thickness skin graft was placed with anticipated postoperative contraction to further shrink the wound. Future placement of tissue expanders and resection of the skin graft will allow for further advancement and wound closure with an improved contour.

**Figure 4:**
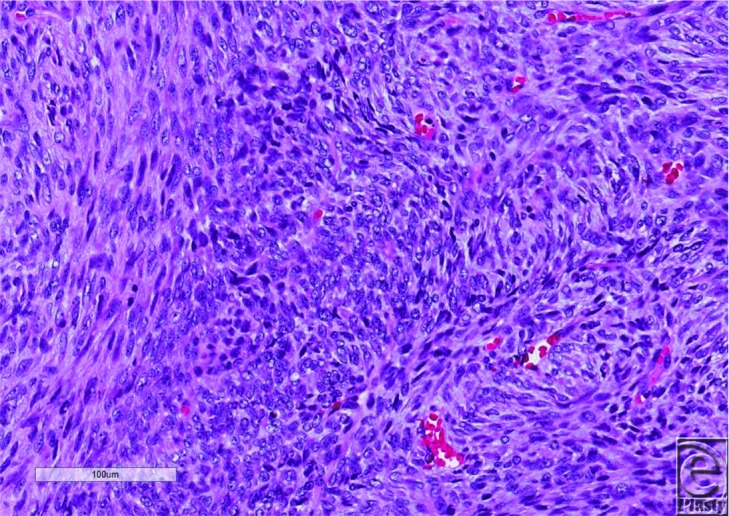
Fibrosarcomatous changes within dermatofibrosarcoma protuberans on final pathological analysis.

DFSP in the head and neck is problematic, and preoperative comorbidities may drive the reconstruction toward less invasive options. Given the usual recommendation for a clear margin of underlying fascia, it is important to plan for these defects. Preoperative discussions with the patient about cosmetic deformity and recurrence are imperative to manage expectations. In our case, the tumor was excised with minimal sacrifice of the anterior rectus fascia with a total defect size of 36 × 33 cm (Figs 1 and 2). Our initial plan for a thigh-based musculocutaneous or fasciocutaneous flap was aborted, given the limited disruption of the abdominal wall. We elected to perform advancement of abdominal tissue, which decreased the wound size to 17 × 10 cm. Split-thickness skin grafting was performed to utilize the inherent contraction over time to further decrease the size of the defect (Fig 3). Upon histopathological examination of the tumor, it was found to be myxoid DFSP with fibrosarcomatous changes (Fig 4). Our future plans of tissue expansion and further advancement of the abdominal flaps for a uniform contour of the abdominal wall will ultimately provide the patient with an acceptable cosmetic result.

DFSP is a rare and slow and asymmetrically growing tumor of the dermis and subcutaneous tissue.^1^^-^3 Diagnosis is often delayed because of the initially benign-appearing course. Historically, recurrence rates have been high and wide excisions with margins were the standard. Recent studies have shown pathological analysis by an experienced dermatopathologist to be just as important and that margins of 1 to 2 cm may be acceptable.^2^ Patients with fibrosarcomatous changes should be more closely monitored, as these have a risk for distant metastasis. Preoperative discussions should be thorough to help manage expectations about recurrence and deformities. A multitude of reconstructive options should be considered preoperatively as the ultimate defect can be unpredictable.
